# Diaqua­bis(5-carb­oxy-2-methyl-1*H*-imidazole-4-carboxyl­ato-κ^2^ *N* ^3^,*O* ^4^)manganese(II)

**DOI:** 10.1107/S160053680800411X

**Published:** 2008-02-15

**Authors:** Jian-Zhong Zeng, Xiu-Guang Yi, Jun-Yue Lin, Shao-Ming Ying, Gan-Sheng Huang

**Affiliations:** a College of Life Sciences, Jinggangshan University, Ji’an, Jiangxi 343009, People’s Republic of China; b College of Chemistry and Chemical Engineering, Jinggangshan University, Ji’an, Jiangxi 343009, People’s Republic of China

## Abstract

The title complex, [Mn(C_6_H_5_N_2_O_4_)_2_(H_2_O)_2_], was obtained by hydro­thermal synthesis. The Mn^II^ atom, which lies on an inversion centre, displays a slightly distorted octa­hedral geometry. In the crystal packing, complex mol­ecules are linked by inter­molecular O—H⋯O and N—H⋯O hydrogen bonds to form a three-dimensional supramolecular structure. The title complex is isostructural with the corresponding cadmium(II) complex [Nie, Wen, Wu, Liu & Liu (2007 ▶). *Acta Cryst.* E**63**, m753–m755].

## Related literature

For related literature, see: Liang *et al.* (2002 ▶); Net *et al.* (1989 ▶); Nie *et al.* (2007 ▶); Ying & Mao (2006 ▶).
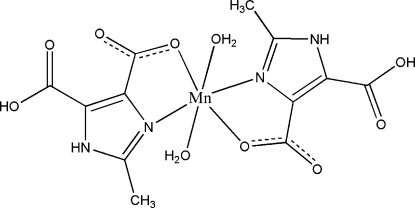


## Experimental

### 

#### Crystal data

[Mn(C_6_H_5_N_2_O_4_)_2_(H_2_O)_2_]
*M* *_r_* = 429.21
Monoclinic, 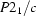

*a* = 12.2047 (12) Å
*b* = 9.1607 (9) Å
*c* = 7.3860 (7) Å
β = 101.355 (2)°
*V* = 809.62 (14) Å^3^
*Z* = 2
Mo *K*α radiation
μ = 0.88 mm^−1^
*T* = 293 (2) K
0.30 × 0.21 × 0.12 mm

#### Data collection

Bruker APEX area-detector diffractometer
Absorption correction: multi-scan (*SADABS*; Sheldrick, 2002 ▶) *T*_min_ = 0.778, *T*_max_ = 0.902
5936 measured reflections
1931 independent reflections
1387 reflections with *I* > 2σ(*I*)
*R*_int_ = 0.033

#### Refinement

*R*[*F* ^2^ > 2σ(*F* ^2^)] = 0.033
*wR*(*F* ^2^) = 0.096
*S* = 0.96
1931 reflections
132 parameters
H atoms treated by a mixture of independent and constrained refinement
Δρ_max_ = 0.35 e Å^−3^
Δρ_min_ = −0.35 e Å^−3^

### 

Data collection: *SMART* (Bruker, 2004 ▶); cell refinement: *SAINT* (Bruker, 2004 ▶); data reduction: *SAINT*; program(s) used to solve structure: *SHELXS97* (Sheldrick, 2008 ▶); program(s) used to refine structure: *SHELXL97* (Sheldrick, 2008 ▶); molecular graphics: *SHELXTL* (Version 5.1; Sheldrick, 2008 ▶); software used to prepare material for publication: *SHELXTL*.

## Supplementary Material

Crystal structure: contains datablocks I, global. DOI: 10.1107/S160053680800411X/rz2194sup1.cif

Structure factors: contains datablocks I. DOI: 10.1107/S160053680800411X/rz2194Isup2.hkl

Additional supplementary materials:  crystallographic information; 3D view; checkCIF report

## Figures and Tables

**Table 1:** Hydrogen-bond geometry (Å, °)

*D*—H⋯*A*	*D*—H	H⋯*A*	*D*⋯*A*	*D*—H⋯*A*
O5—H5*B*⋯O3^i^	0.77 (3)	2.01 (3)	2.763 (2)	168 (3)
O5—H5*A*⋯O4^ii^	0.78 (3)	1.98 (3)	2.760 (2)	174 (3)
N2—H2*A*⋯O1^iii^	0.86	2.06	2.841 (2)	151

Symmetry codes: (i) 
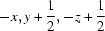
; (ii) 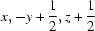; (iii) 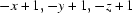.

**Fig. 1.**
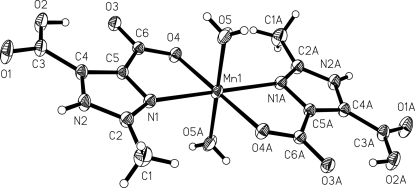
The molecular structure of the title compound with displacement ellipsoids drawn at the 30% probability level. Symmetry code: (A) -x, -y + 1, -z.

**Fig. 2.**
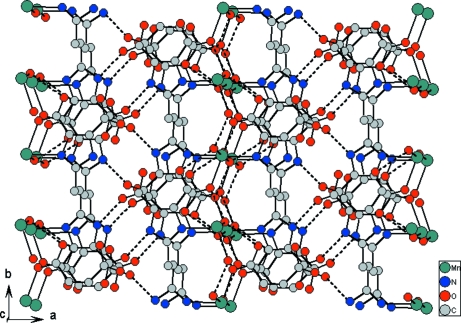
Packing diagram of the title compound viewed along the c axis. Hydrogen atoms are omitted for clarity. The hydrogen bonds are drawn as dotted lines.

## supplementary crystallographic information

### Comment

The use of multifunctional ligands to construct coordination polymers is of current interest due to their potential ability to generate new solid materials with novel network topologies by deliberate design (Ying & Mao, 2006). In these studies, much attention has been put into coordination polymers containing metals and N-heterocyclic carboxylic acids because they can exhibit abundant structural type and can be potentially used as functional materials (Nie *et al.*, 2007; Liang *et al.*, 2002; Net *et al.*, 1989). In this paper, we report the synthesis and structure of a new manganese(II) complex obtained from 2-methyl-1*H*-imidazole-4,5-dicarboxylic acid (H_3_MIA).

The title mononuclear complex molecule contains one manganese(II) ion, two mono-deprotonated H_2_MIA ligands and two water molecules. The manganese(II) ion lies on an inversion centre and is six-coordinated by two carboxylate oxygen atoms and two nitrogen atoms of the H_2_MIA ligands, and by the oxygen atoms of two water molecules forming a slightly distorted octahedral geometry (Fig. 1). The Mn—O distances are 2.1433 (19) and 2.2103 (13) Å and the Mn—N distance is 2.2700 (16) Å. In the crystal packing, complex molecules are linked by intermolecular O—H···O and N—H···O hydrogen bonds to form a three-dimensional supermolecular structure (Fig. 2). The complex is isostructural with the corresponding cadmium(II) complex which has been reported recently (Nie *et al.*, 2007).

### Experimental

A mixture of manganese(II) acetate (0.5 mmol, 0.120 g) and 2-methyl-1*H*-imidazole-4,5-dicarboxylic acid in 10 ml of distilled water was sealed in an autoclave equipped with a Teflon liner (20 ml) and then heated at 150°C for 3 days. Crystals of the title compound were obtained by slow evaporation of the solvent at room temperature.

### Refinement

The water H atoms were located in a difference Fourier map and refined freely, with *U*_iso_(H) = 1.5 *U*_eq_(O). All other H atoms were positioned geometrically and refined in the riding-model approximation, with C—H = 0.97 Å, N—H = 0.86 Å, O—H = 0.82 Å and with *U*_iso_(H) = 1.2 *U*_eq_(N) or 1.5 *U*_eq_(C, O)

### Crystal data

**Table:**

[Mn(C_6_H_5_N_2_O_4_)_2_(H_2_O)_2_]	*F*_000_ = 438
*M**_r_* = 429.21	*D*_x_ = 1.761 Mg m^−^^3^
Monoclinic, *P*2_1_/*c*	Mo *K*α radiation λ = 0.71073 Å
Hall symbol: -P 2ybc	Cell parameters from 3147 reflections
*a* = 12.2047 (12) Å	θ = 2.8–28.2º
*b* = 9.1607 (9) Å	µ = 0.88 mm^−^^1^
*c* = 7.3860 (7) Å	*T* = 293 (2) K
β = 101.355 (2)º	Plate, colourless
*V* = 809.62 (14) Å^3^	0.30 × 0.21 × 0.12 mm
*Z* = 2	

### Data collection

**Table:**

Bruker APEX area-detector diffractometer	1931 independent reflections
Radiation source: fine-focus sealed tube	1387 reflections with *I* > 2σ(*I*)
Monochromator: graphite	*R*_int_ = 0.033
*T* = 293(2) K	θ_max_ = 28.3º
φ and ω scans	θ_min_ = 2.8º
Absorption correction: multi-scan(SADABS; Sheldrick, 2002)	*h* = −16→16
*T*_min_ = 0.778, *T*_max_ = 0.902	*k* = −12→12
5936 measured reflections	*l* = −9→9

### Refinement

**Table:**

Refinement on *F*^2^	Secondary atom site location: difference Fourier map
Least-squares matrix: full	Hydrogen site location: inferred from neighbouring sites
*R*[*F*^2^ > 2σ(*F*^2^)] = 0.033	H atoms treated by a mixture of independent and constrained refinement
*wR*(*F*^2^) = 0.096	*w* = 1/[σ^2^(*F*_o_^2^) + (0.0578*P*)^2^] where *P* = (*F*_o_^2^ + 2*F*_c_^2^)/3
*S* = 0.96	(Δ/σ)_max_ < 0.001
1931 reflections	Δρ_max_ = 0.35 e Å^−^^3^
132 parameters	Δρ_min_ = −0.35 e Å^−^^3^
Primary atom site location: structure-invariant direct methods	Extinction correction: none

### Special details

**Table:**

Geometry. All e.s.d.'s (except the e.s.d. in the dihedral angle between two l.s. planes) are estimated using the full covariance matrix. The cell e.s.d.'s are taken into account individually in the estimation of e.s.d.'s in distances, angles and torsion angles; correlations between e.s.d.'s in cell parameters are only used when they are defined by crystal symmetry. An approximate (isotropic) treatment of cell e.s.d.'s is used for estimating e.s.d.'s involving l.s. planes.
Refinement. Refinement of *F*^2^ against ALL reflections. The weighted *R*-factor *wR* and goodness of fit *S* are based on *F*^2^, conventional *R*-factors *R* are based on *F*, with *F* set to zero for negative *F*^2^. The threshold expression of *F*^2^ > σ(*F*^2^) is used only for calculating *R*-factors(gt) *etc*. and is not relevant to the choice of reflections for refinement. *R*-factors based on *F*^2^ are statistically about twice as large as those based on *F*, and *R*- factors based on ALL data will be even larger.

### Fractional atomic coordinates and isotropic or equivalent isotropic displacement parameters (Å^2^)

**Table:**

	*x*	*y*	*z*	*U*_iso_*/*U*_eq_	
Mn1	0.0000	0.5000	0.0000	0.02937 (16)	
N1	0.18504 (13)	0.50974 (16)	0.1269 (2)	0.0293 (4)	
N2	0.35369 (14)	0.52117 (17)	0.2952 (3)	0.0363 (4)	
H2A	0.4143	0.5577	0.3581	0.044*	
O1	0.50357 (12)	0.30235 (19)	0.4426 (3)	0.0559 (5)	
O2	0.39335 (12)	0.12886 (16)	0.3029 (2)	0.0484 (4)	
H2B	0.3298	0.1226	0.2415	0.073*	
O3	0.19549 (11)	0.11771 (14)	0.1132 (2)	0.0352 (4)	
O4	0.06082 (11)	0.27347 (14)	−0.01036 (19)	0.0330 (3)	
O5	−0.02904 (16)	0.4590 (2)	0.2723 (3)	0.0459 (5)	
C1	0.2586 (2)	0.7599 (2)	0.2185 (4)	0.0549 (7)	
H1A	0.1875	0.7922	0.1503	0.082*	
H1B	0.3172	0.8005	0.1645	0.082*	
H1C	0.2675	0.7915	0.3444	0.082*	
C2	0.26397 (16)	0.5981 (2)	0.2124 (3)	0.0339 (5)	
C3	0.41682 (17)	0.2662 (2)	0.3431 (3)	0.0370 (5)	
C4	0.33359 (15)	0.3766 (2)	0.2638 (3)	0.0309 (5)	
C5	0.22732 (15)	0.3710 (2)	0.1569 (3)	0.0273 (4)	
C6	0.15691 (15)	0.2449 (2)	0.0818 (3)	0.0277 (4)	
H5B	−0.071 (2)	0.499 (2)	0.319 (4)	0.041 (8)*	
H5A	0.001 (2)	0.397 (3)	0.336 (4)	0.065 (9)*	

### Atomic displacement parameters (Å^2^)

**Table:**

	*U*^11^	*U*^22^	*U*^33^	*U*^12^	*U*^13^	*U*^23^
Mn1	0.0254 (2)	0.0274 (2)	0.0312 (3)	0.00137 (17)	−0.00438 (17)	0.00092 (17)
N1	0.0265 (9)	0.0253 (8)	0.0328 (10)	−0.0015 (6)	−0.0023 (7)	−0.0002 (7)
N2	0.0232 (8)	0.0327 (10)	0.0468 (12)	−0.0040 (7)	−0.0081 (7)	−0.0053 (8)
O1	0.0338 (9)	0.0507 (10)	0.0697 (12)	0.0059 (7)	−0.0231 (8)	−0.0116 (9)
O2	0.0350 (8)	0.0358 (9)	0.0650 (11)	0.0064 (7)	−0.0127 (7)	−0.0026 (7)
O3	0.0305 (7)	0.0248 (7)	0.0466 (9)	−0.0008 (6)	−0.0011 (6)	−0.0008 (6)
O4	0.0275 (7)	0.0276 (7)	0.0379 (9)	−0.0018 (6)	−0.0080 (6)	−0.0034 (6)
O5	0.0493 (11)	0.0499 (10)	0.0391 (10)	0.0240 (9)	0.0099 (8)	0.0137 (8)
C1	0.0500 (15)	0.0375 (13)	0.071 (2)	0.0001 (11)	−0.0042 (13)	−0.0063 (12)
C2	0.0283 (10)	0.0309 (10)	0.0388 (13)	−0.0037 (8)	−0.0029 (9)	−0.0022 (9)
C3	0.0276 (10)	0.0376 (12)	0.0415 (14)	0.0043 (8)	−0.0037 (9)	−0.0016 (10)
C4	0.0244 (10)	0.0317 (11)	0.0335 (12)	−0.0012 (8)	−0.0022 (8)	−0.0025 (8)
C5	0.0230 (9)	0.0293 (10)	0.0275 (11)	−0.0010 (7)	−0.0003 (7)	−0.0006 (8)
C6	0.0255 (10)	0.0276 (10)	0.0286 (11)	−0.0023 (8)	0.0015 (8)	−0.0010 (8)

### Geometric parameters (Å, °)

**Table:**

Mn1—O5^i^	2.1433 (19)	O2—H2B	0.8200
Mn1—O5	2.1433 (19)	O3—C6	1.261 (2)
Mn1—O4	2.2103 (13)	O4—C6	1.262 (2)
Mn1—O4^i^	2.2103 (13)	O5—H5B	0.77 (3)
Mn1—N1	2.2700 (16)	O5—H5A	0.78 (3)
Mn1—N1^i^	2.2700 (16)	C1—C2	1.485 (3)
N1—C2	1.320 (2)	C1—H1A	0.9600
N1—C5	1.373 (2)	C1—H1B	0.9600
N2—C2	1.344 (3)	C1—H1C	0.9600
N2—C4	1.358 (2)	C3—C4	1.471 (3)
N2—H2A	0.8600	C4—C5	1.380 (3)
O1—C3	1.210 (2)	C5—C6	1.481 (3)
O2—C3	1.311 (2)		
			
O5^i^—Mn1—O5	180.00 (10)	Mn1—O5—H5A	124 (2)
O5^i^—Mn1—O4	90.77 (6)	H5B—O5—H5A	110 (3)
O5—Mn1—O4	89.23 (6)	C2—C1—H1A	109.5
O5^i^—Mn1—O4^i^	89.23 (6)	C2—C1—H1B	109.5
O5—Mn1—O4^i^	90.77 (6)	H1A—C1—H1B	109.5
O4—Mn1—O4^i^	180.00 (10)	C2—C1—H1C	109.5
O5^i^—Mn1—N1	92.62 (7)	H1A—C1—H1C	109.5
O5—Mn1—N1	87.38 (7)	H1B—C1—H1C	109.5
O4—Mn1—N1	74.79 (5)	N1—C2—N2	110.42 (18)
O4^i^—Mn1—N1	105.21 (5)	N1—C2—C1	126.44 (19)
O5^i^—Mn1—N1^i^	87.38 (7)	N2—C2—C1	123.14 (18)
O5—Mn1—N1^i^	92.62 (7)	O1—C3—O2	121.74 (19)
O4—Mn1—N1^i^	105.21 (5)	O1—C3—C4	120.44 (19)
O4^i^—Mn1—N1^i^	74.79 (5)	O2—C3—C4	117.82 (17)
N1—Mn1—N1^i^	180.00 (8)	N2—C4—C5	104.59 (16)
C2—N1—C5	105.93 (16)	N2—C4—C3	121.00 (17)
C2—N1—Mn1	142.63 (14)	C5—C4—C3	134.39 (18)
C5—N1—Mn1	110.00 (11)	N1—C5—C4	109.80 (15)
C2—N2—C4	109.25 (16)	N1—C5—C6	119.32 (16)
C2—N2—H2A	125.4	C4—C5—C6	130.84 (17)
C4—N2—H2A	125.4	O4—C6—O3	124.39 (16)
C3—O2—H2B	109.5	O4—C6—C5	116.68 (16)
C6—O4—Mn1	117.32 (11)	O3—C6—C5	118.93 (16)
Mn1—O5—H5B	126 (2)		

Symmetry codes: (i) −*x*, −*y*+1, −*z*.

### Hydrogen-bond geometry (Å, °)

**Table:**

*D*—H···*A*	*D*—H	H···*A*	*D*···*A*	*D*—H···*A*
O5—H5B···O3^ii^	0.77 (3)	2.01 (3)	2.763 (2)	168 (3)
O5—H5A···O4^iii^	0.78 (3)	1.98 (3)	2.760 (2)	174 (3)
N2—H2A···O1^iv^	0.86	2.06	2.841 (2)	151

Symmetry codes: (ii) −*x*, *y*+1/2, −*z*+1/2; (iii) *x*, −*y*+1/2, *z*+1/2; (iv) −*x*+1, −*y*+1, −*z*+1.
